# Choosing the Right Non-Statin Therapy for the Right Patient – How To Sequence Advanced Lipid-Lowering Therapies

**DOI:** 10.1007/s11883-026-01390-7

**Published:** 2026-02-26

**Authors:** Julius L. Katzmann, Ulrich Laufs

**Affiliations:** Klinik und Poliklinik für Kardiologie, Universitätsklinikum Leipzig, Liebigstraße 20, Leipzig, 04103 Germany

**Keywords:** Triglycerides, Lipoprotein(a), PCSK9 inhibitor, CETP inhibitor, ANGPTL3 inhibition, ApoCIII inhibition

## Abstract

**Purpose of Review:**

On the basis of life-style changes and statins, current guidelines recommend early combination therapy to reduce LDL cholesterol (LDL-C). Available and future novel non-statin lipid-lowering therapies may have specific advantages in patients with (1) statin intolerance, (2) elevated triglyceride-rich lipoproteins, (3) elevated lipoprotein(a), and (4) rare genetic dyslipidemias.

**Recent Findings:**

Currently available treatment options to lower LDL-C with proven cardiovascular benefit include statins, ezetimibe, bempedoic acid, and PCSK9 antibodies. The 2025 update of the ESC/EAS dyslipidemia guidelines incorporates recommendations on early combination treatment and management of rare dyslipidemias, which are detailed in this review. Novel LDL-C-lowering strategies, targeting PCSK9 and CETP, may further improve dyslipidemia management. Drugs in development with profound effects on lipoprotein(a) or triglyceride concentration may allow for specific modification of residual cardiovascular risk. Innovative DNA-targeting therapies are moving towards clinical testing in larger studies.

**Summary:**

Various treatment options for patients with dyslipidemia and distinct characteristics have become available. Future developments may allow for even more tailored treatment, depending on dyslipidemia phenotype.

## Introduction

Low-density lipoprotein cholesterol (LDL-C) is the primary target in dyslipidemia management. To reduce the risk of first or recurrent cardiovascular events, statins remain the cornerstone of lipid-lowering treatment. In the recent years, three additional options with proven cardiovascular benefit have become available, i.e., ezetimibe, bempedoic acid, and the PCSK9 monoclonal antibodies alirocumab and evolocumab. Several novel LDL-C-lowering drugs are at earlier stages of development. Combination of these drugs allows for lowering of LDL-C to concentrations not achievable with statins alone, further reductions in cardiovascular risk, and for tailored approaches in specific patient cohorts, such as those with statin intolerance. Beyond LDL-C-lowering approaches, novel drugs addressing residual cardiovascular risk by lowering elevated triglycerides or lipoprotein(a) [Lp(a)] have been developed and may further improve dyslipidemia management in the near future.

The recently updated ESC/EAS guidelines on the management of dyslipidemias incorporate important novel recommendations on early combination lipid-lowering treatment after acute coronary syndrome (ACS) and the management of rare dyslipidemias [1, 2]. In this review, these recommendations and the rationale for non-statin lipid-lowering treatment are presented, followed by details on currently available and future treatment options (Table 1).

**Table 1:** Non-statin lipid-lowering drugs

Target	Drug	Substance class, route of administration	Efficacy	Evidence status
NPC1L1	Ezetimibe	Small molecule, oral	LDL-C 20–25% ↓	Positive outcomes trial (IMPROVE-IT [4])
ATP citrate lyase	Bempedoic acid	Small molecule, oral	LDL-C 16–25% ↓	Positive outcomes trial (CLEAR Outcomes [8])
PCSK9	Evolocumab	Fully human monoclonal antibody, s. c.	LDL-C 60% ↓	Two positive outcomes trials (FOURIER [5], VESALIUS-CV [6])
	Alirocumab	Fully human monoclonal antibody, s. c.	LDL-C 60% ↓	Positive outcomes trial (ODYSSEY Outcomes [7])
	Inclisiran	Small-interfering RNA, s. c.	LDL-C 50% ↓	Two outcomes trials ongoing (ORION-4, VICTORION-2 PREVENT)
	Recaticimab	Humanized monoclonal antibody, s. c.	LDL-C 50–55% ↓	Phase 3 trials completed [51]
	Lerodalcibep	Fusion protein, s. c.	LDL-C 55–60% ↓	Phase 3 trials completed [52]
	Enlicitide (formerly MK-0616)	Macrocyclic peptide, oral	LDL-C 60% ↓	Outcomes trial ongoing (CORALreef Outcomes)
	Laroprovstat (formerly AZD0780)	Small molecule, oral	LDL-C 55–60% ↓	Outcomes trial ongoing (AZURE Outcomes)
CETP	Obicetrapib	Small molecule, oral	LDL-C 45–50% ↓	Outcomes trial ongoing (PREVAIL)
Lipoprotein(a)	Pelacarsen	Antisense oligonucleotide, s. c.	Lipoprotein(a) 80% ↓	Outcomes trial ongoing (Lp(a)-HORIZON)
	Lepodisiran	Small-interfering RNA, s. c.	Lipoprotein(a) 95% ↓	Outcomes trial ongoing (ACCLAIM-Lp(a))
	Olpasiran	Small-interfering RNA, s. c.	Lipoprotein(a) 95% ↓	Outcomes trial ongoing (OCEAN(a)-Outcomes Trial)
	Zerlasiran	Small-interfering RNA, s. c.	Lipoprotein(a) 85% ↓	Outcomes trial announced
	Muvalaplin	Small molecule, oral	Lipoprotein(a) 65–85% ↓	Outcomes trial ongoing (MOVE-Lp(a))
ANGPTL3	Evinacumab	Fully human monoclonal antibody, i. v.	LDL-C 50% ↓ in HoFH	Phase 3 trials in HoFH completed [48]
	SHR-1918	Fully human monoclonal antibody, s. c.	LDL-C up to ~ 30% ↓ Triglycerides 50% ↓	Phase 2 trial completed [70]
	Zodasiran (formerly ARO-ANG3)	Small-interfering RNA, s. c.	LDL-C ~ 20% ↓ Triglyceride ~ 50–60% ↓	Phase 3 trial in HoFH ongoing (YOSEMITE)
	Solbinsiran	Small-interfering RNA, s. c.	LDL-C ~ 17% ↓ Triglycerides ~ 50% ↓	Phase 2 trial completed (PROLONG-ANG3 [72])
Apolipoprotein CIII	Volanesorsen	Antisense oligonucleotide, s. c.	Triglycerides up to 77% ↓ in FCS	Phase 3 trials completed [49, 50]
	Olezarsen	Antisense oligonucleotide, s. c.	Triglycerides 50–60% ↓	Phase 3 trials completed [75–77]
	Plozasiran	Small-interfering RNA, s. c.	Triglycerides 60% ↓	Phase 3 trials completed, cardiovascular outcomes trial announced (CAPITAN)

Abbreviations: ↓: reduction, *FCS* familial chylomicronemia syndrome, *HoFH* homozygous familial hypercholesterolemia, *i.v.* intravenous, *LDL-C* low-density lipoprotein cholesterol, *s.c.* subcutaneous

## Rationale for Non-Statin Lipid-Lowering Therapies

### Incremental Cardiovascular Benefit of Further LDL-C-lowering

**Fig. 1:**
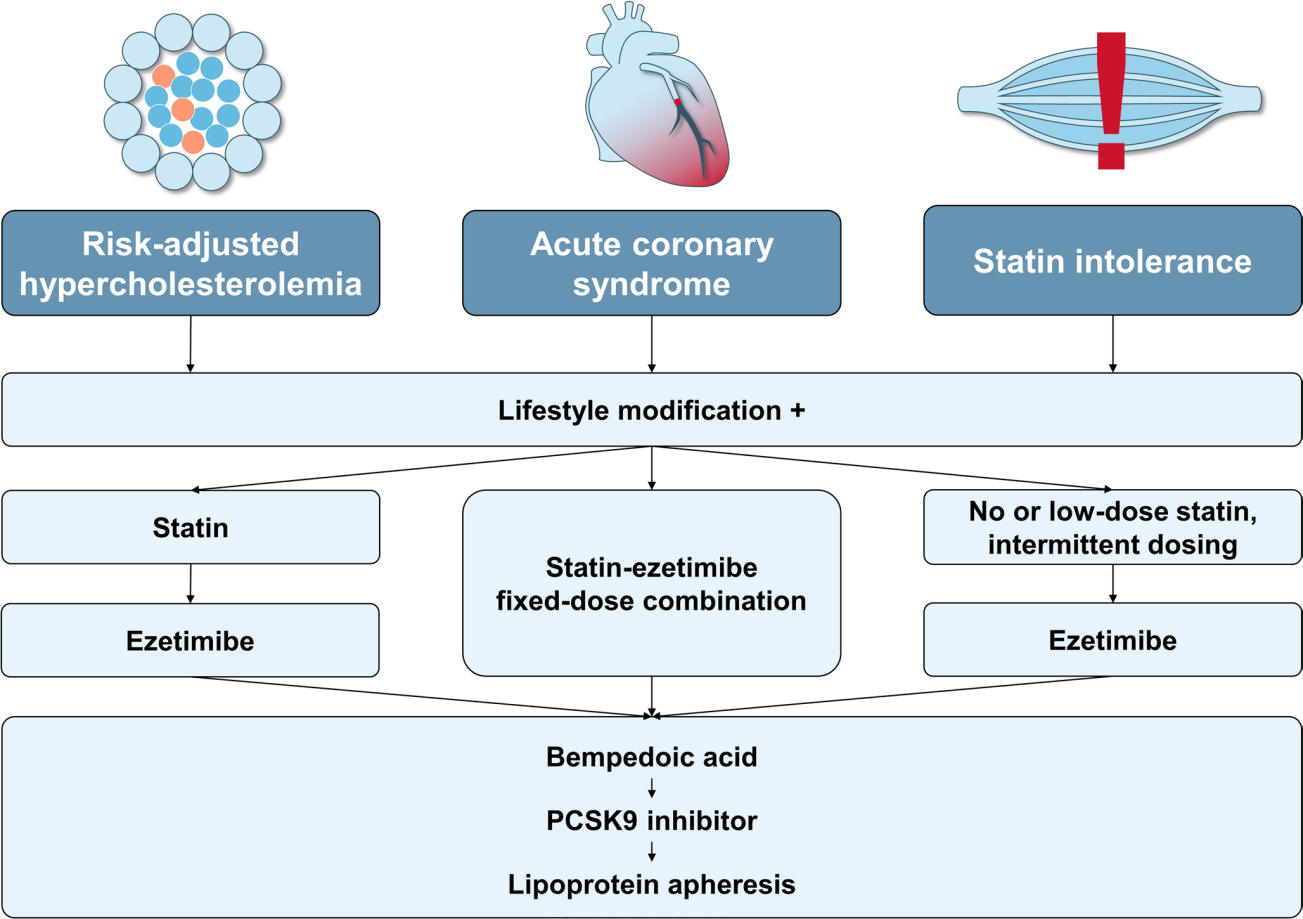
Suggested treatment algorithms for hypercholesterolemia in different patient populations

The benefits of statin therapy have been documented for decades [3]. Therefore, statin therapy remains the cornerstone in dyslipidemia management [2]. With the IMPROVE-IT study for ezetimibe [4], the FOURIER [5] and the VESALIUS-CV [6] trials with evolocumab, the ODYSSEY Outcomes trial with alirocumab [7], and the CLEAR Outcomes trial with bempedoic acid [8], four agents have shown additional cardiovascular benefits in patients on statin or with statin intolerance by further reducing LDL-C. Treatment with these agents allowed for the achievement of lower LDL-C concentrations than achievable with statin monotherapy, and represented the rationale for the revised LDL-C targets in the 2019 ESC/EAS dyslipidemia guidelines [2], which, of note, have remained unchanged in the 2025 dyslipidemia guidelines update [1]. A possible treatment algorithm for hypercholesterolemia with these drugs is depicted in Fig. 1.

### Statin Intolerance

The inability to tolerate statin therapy beyond low-dose statins, or statin therapy at all, has been referred to as “statin intolerance”. The most frequently reported symptoms encompass muscle symptoms, however n-of-1 trials indicate that 90% of the symptoms can be attributed to nocebo effects [9]. Nonetheless, statin intolerance is a frequent phenomenon that affects approximately 9% of patients on statin [10], and has been associated with increased cardiovascular risk [11], increased healthcare costs [12], low quality of life [13], and low rates of LDL-C target attainment [14]. As these patients are frequently unable or unwilling to take a statin, there is a clear need for other medications for reducing LDL-C. The treatment algorithm for these patients should include attempts to establish the highest tolerable dose of a statin and the addition of non-statins to reach the individual LDL-C goals (Fig. 1).

### Residual Risk Attributed To Elevated Triglycerides or Lipoprotein(a)

**Fig. 2:**
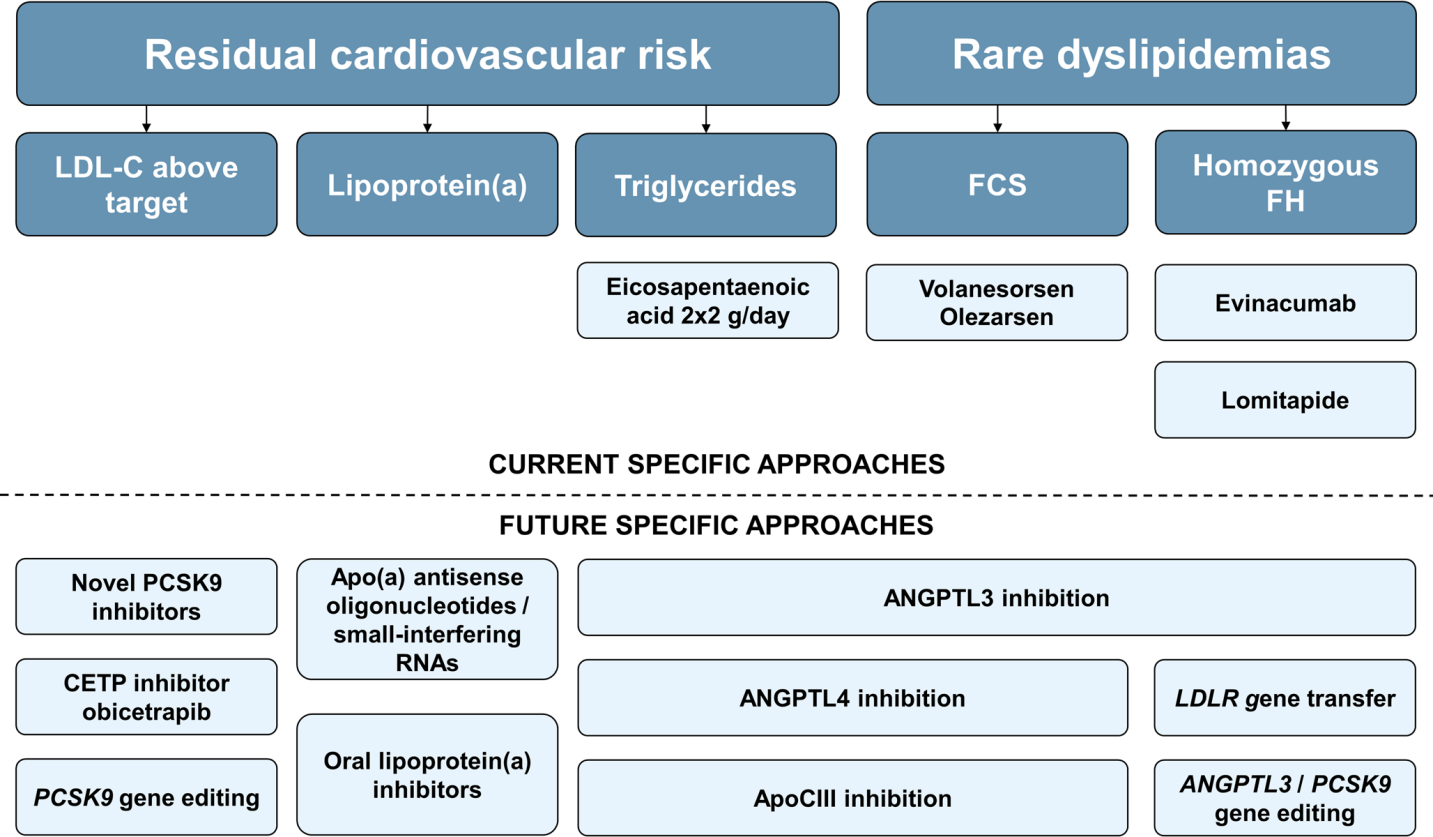
Current and future approaches for the treatment of residual cardiovascular risk and rare dyslipidemias

Despite controlled LDL-C, patients may face progression of atherosclerosis. This can be attributed to other uncontrolled cardiovascular risk factors such as hypertension or diabetes, or non-LDL dyslipidemias with elevated triglyceride-rich lipoproteins or Lp(a). As statins do not specifically address either, non-statin options are needed in these patients (Fig. 2).

In the past, several attempts to lower triglycerides with fibrates have failed to demonstrate cardiovascular benefit on top of statins, with the latest example being the highly potent and specific PPARα modulator pemafibrate. This agent was tested in the PROMINENT study of 10,497 patients with type 2 diabetes, elevated triglycerides, and low HDL cholesterol. Despite a 26% triglyceride reduction, no effect on cardiovascular risk was observed, potentially due to the lack of reductions in the total concentration of atherogenic lipoproteins expressed as apolipoprotein B concentration, which increased by 5% [15]. Therefore, fibrates are not recommended to reduce cardiovascular risk [1].

A successful pharmacologic intervention in patients with elevated triglycerides was demonstrated in the REDUCE-IT study with high-dose eicosapentaenoic acid (EPA). EPA treatment led to a 25% relative risk reduction [16]. In contrast, the STRENGTH trial with a mixture of lower doses of EPA with docosahexaenoic acid (DHA) was neutral [17]. Therefore, the 2025 ESC/EAS guidelines update recommends treatment 2 × 2 g/d EPA in patients with hypertriglyceridemia and high or very-high cardiovascular risk [1].

Several agents for selective and potent lowering of Lp(a) are tested in cardiovascular outcome trials (Sect. 4.2). The current strategy in patients with Lp(a) elevation is to modify all other cardiovascular risk factors to reduce global cardiovascular risk [18], with a special focus on LDL-C lowering. Registry data suggest that optimal LDL-C lowering is associated with significantly reduced Lp(a)-associated cardiovascular risk [19].

## Current Therapeutic Approaches

### Statins, Ezetimibe, and Statin-Ezetimibe Combinations

Lifestyle modification is the basis of therapy in treating dyslipidemia. While lifestyle modification is highly effective in reducing elevated triglycerides [20], the effects on LDL-C concentration are usually small (< 10% [2]). The most important components of lifestyle modification are smoking cessation and physical exercise. The positive effects of these interventions are not reflected by major changes in LDL-C.

The LDL-C treatment targets remained unchanged in the 2025 update of the ESC/EAS dyslipidemia guidelines: for patients at very-high or extreme risk, LDL-C targets of < 1.4 mmol/L and < 1.0 mmol/L, respectively, are recommended [1]. As these target concentrations are derived from studies based on combination lipid-lowering therapies, it comes as no surprise that statin monotherapy is usually not sufficient to attain the LDL-C targets. Nevertheless, statin monotherapy still represents the majority of lipid-lowering treatment in many countries [21–24] and hence, the LDL-C targets are only infrequently achieved [23, 25–27]. Based on published efficacy estimates of available lipid-lowering agents, simulation studies suggest that utilizing the available treatment options, > 90% of patients should be able to achieve the LDL-C targets [26, 28, 29].

Several expert opinion papers have called for first-line combination lipid-lowering therapy in patients at very-high risk, including those with ACS [30–32]. The rationale for this approach is manifold. Most importantly, patients post ACS represent a vulnerable population with high risk of recurrent events, and with a stepwise treatment approach, target attainment is delayed for several weeks or even months. An analysis of the SWEDEHEART registry showed that earlier initiation of ezetimibe was associated with reduced cardiovascular risk compared to later or no ezetimibe treatment [33]. Furthermore, randomized studies comparing high-intensity statin vs. moderate-intensity statin and ezetimibe (RACING study [34]), and high-intensity statin therapy vs. a treatment-target approach (LODESTAR study [35]) showed non-inferiority of the combination and treatment-target approaches regarding cardiovascular events with fewer drug discontinuations due to adverse effects, further corroborating the upfront combination therapy approach. Furthermore, a pragmatic reason is the observation that intensification of lipid-lowering treatment is uncommon in general practice. First or recurrent hospitalizations for ACS represent an opportunity for treatment intensification, which is frequently not implemented in ambulatory care [22, 36].

In line with this, the 2025 update of the ESC/EAS dyslipidemia guidelines now endorses a concept of upfront combination lipid-lowering therapy with a statin and ezetimibe in treatment-naïve patients with ACS, in whom target achievement with statin monotherapy, based on the expected LDL-C reduction, is unlikely. Furthermore, patients hospitalized with ACS who were already on lipid-lowering treatment should undergo intensification of treatment to further lower LDL-C [1].

In contrast to the ESC hypertension guidelines, which not only recommend combination therapy, but combination *pills* (“fixed-dose combinations”) since the 2018 guidelines [37], no such recommendation is given in the dyslipidemia guidelines. The evidence base for advantages of single-pill combinations compared to separate pills is less strong for lipid-lowering treatment compared to antihypertensive medication, however retrospective data indicate lower LDL-C concentrations [38] and better medication adherence and persistence for single-pill combinations vs. separate pills [39]. Given their generic availability, the authors recommend the prescription of single-pill combinations of a statin and ezetimibe to reduce pill burden and improve medication adherence and persistence (Fig. 1).

The principles of the ESC/EAS guideline recommendations are generally in line with international recommendations such as the US guidelines on the management of blood cholesterol [40]. Differences exist in the concept of LDL-C treatment targets endorsed by the European guidelines, whereas the US guidelines recommend a threshold-based approach (1.8 mmol/L) without target value for the addition of non-statin drugs in very-high-risk patients. The treatment sequence – statins, ezetimibe, PCSK9 antibodies – does not differ, while no upfront combination therapy is recommended in the US guidelines.

### Bempedoic Acid

Bempedoic acid is the third orally available option for lowering LDL-C besides statins and ezetimibe. In the CLEAR Outcomes trial, 13,970 patients with statin intolerance were randomized to bempedoic acid vs. placebo and followed for a median of 41 months. The baseline LDL-C concentration of 3.6 mmol/L was reduced by 21% at 6 months compared to placebo. The incidence of the primary endpoint of death from cardiovascular causes, non-fatal myocardial infarction, non-fatal stroke, or coronary revascularization was significantly reduced by relative 13% [8]. Similar to statins, bempedoic acid significantly reduced high-sensitivity C-reactive protein. Importantly, the incidence of muscle-related adverse effects was not increased under bempedoic acid (5.6% compared to 6.8% under placebo). Bempedoic acid increases uric acid and the risk of gout.

The 2025 ESC/EAS dyslipidemia guidelines update includes novel recommendations following the CLEAR Outcomes trial, i.e., for treatment with non-statin lipid-lowering agents with proven cardiovascular benefit in patients unable to take a statin in general, and for bempedoic acid in specific for the treatment of patients with statin intolerance. Furthermore, bempedoic acid is recommended as additional treatment to statins and ezetimibe if the LDL-C target is not attained (level of evidence: C) [1].

### PCSK9 Antibodies and Small Interfering RNA

The PCSK9 antibodies evolocumab and alirocumab, and the small interfering RNA inclisiran represent potent options to further lower LDL-C, and have been reviewed in detail before [41]. While for evolocumab and alirocumab, cardiovascular outcomes trials have been completed, showing a 15% relative reduction in risk for both agents [5, 7], two large outcomes trials for inclisiran are currently ongoing (ORION-4, NCT03705234, and VICTORION-2 PREVENT, NCT05030428). VICTORION-Difference, a large phase 4 double-blind, placebo-controlled trial, randomized 1,770 individuals with hypercholesterolemia at high- or very-high cardiovascular risk to receive inclisiran or a rosuvastatin-based individually optimized lipid-lowering therapy (ioLLT). The trial showed that the PCSK9i-based treatment strategy was superior to ioLLT in delivering early and sustained LDL-C reduction, with fewer patient-related outcomes such as muscle-related adverse events, and with better quality of life [42].

Long-term data have become available for evolocumab for up to 8 years of follow-up [43], for alirocumab for up to 3 to 5 years [44], and for inclisiran with maximum exposure of almost 7 years [45]. In all studies, no attenuation of LDL-C-lowering efficacy was found, and the rate of adverse effects was generally low. These data corroborate the important role of these agents especially in patients at the highest risk, those with very high LDL-C level, and those who do not tolerate statin therapy.

The access to PCSK9 inhibitors is limited, mainly because of comparably high treatment costs, and varies among countries [31]. Among the PCSK9 inhibitors, the monoclonal antibodies on average lead to greater reductions in LDL-C of approximately 60%, whereas LDL-C-lowering of inclisiran ranges at approximately 50%, depending on background medication [46]. Inclisiran may have advantages in patients with low medication adherence with injections only every 6 months (after the first two injections within 3 months).

### Drugs Approved for Rare Diseases

Rare, genetically caused dyslipidemias include, among others, homozygous familial hypercholesterolemia (HoFH) and the familial chylomicronemia syndrome (FCS).

HoFH is characterized by extremely elevated LDL-C due to a partial or complete lack of LDL receptor function, which markedly reduces efficacy of LDL receptor-dependent agents (statins, ezetimibe, bempedoic acid, PCSK9 inhibitors). Treatment options include LDL receptor-independent compounds such as evinacumab and lomitapide, and LDL apheresis [47]. The 2025 ESC/EAS dyslipidemia guidelines include a novel recommendation for the treatment with the monoclonal antibody evinacumab. Evinacumab targets angiopoietin-like 3 (ANGPTL3) and reduces LDL-C concentration by approximately 50%, independently of residual LDL receptor function [1, 48].

The FCS is caused by a lack of lipoprotein lipase function and characterized by substantial elevations of triglycerides and high risk of (recurrent) pancreatitis. The 2025 ESC/EAS dyslipidemia guidelines update includes a recommendation for treatment with volanesorsen, an apolipoprotein CIII (apoCIII) antisense oligonucleotide (ASO) [1]. Volanesorsen reduces triglyceride level in FCS patients by up to 77% [49], and in a meta-analysis, a significant reduction in the risk of pancreatitis has been shown [50]. However, treatment is limited by frequent injection site reactions and thrombocytopenia as adverse event, and the drug is only approved in Europe. Recently, the apoCIII inhibitor olezarsen – which differs from volanesorsen by *N*-acetylgalactosamine (GalNAc)-mediated uptake in the hepatocytes, which allows for lower dosing, associated with less frequent adverse events – has been approved by the FDA and EMA. New agents with more favorable side effect profile are under investigation [20] (Fig. 2).

## Future Therapeutic Approaches

### LDL-C-Lowering Drugs

#### Novel Injectable PCSK9 Inhibitors

The humanized monoclonal antibody recaticimab has been approved in China. In a phase 3 trial, 450 mg of recaticimab every 3 months reduced LDL-C by 50–55% [51]. In contrast to the fully human antibodies, antidrug antibodies and neutralizing antibodies have been reported in a significant number of patients.

Lerodalcibep is a fusion protein of a small binding protein (“adnectin”) and human serum albumin to extend its half-life, that binds PCSK9 and blocks its interaction with LDL receptors. It is administered subcutaneously (300 mg in 1.2 mL monthly) and is formulated for ambient stability. Lerodalcibep reduced LDL-C by approximately 55–60% [52].

#### Novel Oral PCSK9 Inhibitors

The macrocyclic peptide enlicitide (formerly MK-0616) binds to the PCSK9 catalytic domain, blocking its interaction with the LDL receptor EGF domain, achieving antibody-like LDL-C lowering potency and selectivity. The coupling with a permeability enhancer enables once-daily oral dosing in the fasting state. In a phase 2 trial, enlicitide reduced LDL-C by ~ 60% and was well tolerated, with no dose-dependent adverse events [53, 54]. Enlicitide is tested in the CORALreef Outcomes trial (NCT06008756) enrolling ~ 14,550 patients with or at high risk for atherosclerotic cardiovascular disease (ASCVD) events. The primary endpoint is a composite of coronary heart disease death, myocardial infarction, ischemic stroke, acute limb ischemia or major amputation, or urgent arterial revascularization. The estimated duration of the trial is 6 years.

Laroprovstat (formerly AZD0780) is an oral small-molecule inhibitor. It binds a pocket in the PCSK9 C-terminal domain, inhibiting lysosomal trafficking of PCSK9-LDL receptor complexes and preventing PCSK9-induced LDL receptor degradation without disrupting PCSK9-LDL receptor binding. It is taken once daily, independent of food. Laroprovstat lowered LDL-C by ~ 50% and was well tolerated, with no dose-related adverse events [55]. Ongoing phase 3 trials include a study in those with or at high risk for ASCVD (NCT07000123) and a study in heterozygous FH (NCT07000136). The AZURE Outcomes trial (NCT07000357) is enrolling ~ 15,100 patients post ACS or at high ASCVD risk. The primary endpoint is a composite of cardiovascular death, myocardial infarction, ischemic stroke, acute limb ischemia or major amputation, or urgent arterial revascularization. The estimated duration of the trial is 4.5 years.

#### PCSK9 Gene-Targeting Therapies

Advances in gene editing have enabled creation of loss-of-function mutations in *PCSK9*, mimicking naturally occurring inactivation seen in population studies. One approach uses a lipid nanoparticle delivering mRNA for a base editor and a guide RNA targeting PCSK9. The translated base editor induces an inactivating mutation, leading to sustained ~ 55% LDL-C reduction in preclinical models lasting over a year [56].

In the first human trial in heterozygous FH, higher doses achieved ~ 45–55% LDL-C reductions but caused aminotransferase elevations and thrombocytopenia, attributed to the lipid nanoparticle [57]. A next-generation candidate links the nanoparticle to GalNAc, improving hepatic targeting and tolerability. Early results in heterozygous FH and premature coronary artery disease show 50–55% LDL-C reductions without significant liver or platelet abnormalities [58].

Another approach, epigenetic editing, silences *PCSK9* via DNA methylation at promoter CpG sites. The editor combines a DNA methyltransferase and transcriptional repressor with a DNA-binding domain, avoiding disruption of DNA sequence. In primates, a single infusion reduced LDL-C by ~ 65% for ≥ 3 months, reversible with a dCas-Tet *PCSK9* activator [59, 60].

The vision of these programs is hat DNA-targeting therapies may one day cure hypercholesterolemia with a single infusion, but clinical programs must confirm long-term safety and rule out off-target effects [61].

#### CETP Inhibitor Obicetrapib

Obicetrapib is an oral cholesteryl ester transfer protein (CETP) inhibitor that enhances HDL-C levels while significantly lowering LDL-C and other atherogenic lipoproteins including Lp(a). It is a next-generation CETP inhibitor designed for high potency with minimal off-target effects compared to earlier agents in this class. In phase 2 trials, oral once-daily obicetrapib (5–10 mg) reduced LDL-C by approximately 45–50% when added to statin therapy and by up to 63% in combination with ezetimibe. It was generally well tolerated, with adverse events similar to placebo and no signal for blood pressure or aldosterone abnormalities seen with prior CETP inhibitors [62, 63]. The phase 3 BROADWAY and BROOKLYN trials have demonstrated LDL-C lowering and safety in patients with ASCVD or heterozygous FH [64]. The large PREVAIL trial (NCT05202509) is assessing cardiovascular outcomes in ~ 9,000 high-risk patients over ~ 4 years.

### Emerging Therapies Targeting Lipoprotein(a)

Pelacarsen is an ASO that reduces hepatic production of apolipoprotein(a), thereby lowering Lp(a) levels. In phase 2 studies, pelacarsen achieved very potent dose-dependent reductions in Lp(a) concentrations. It is administered subcutaneously once monthly and has demonstrated a favorable safety profile to date [65]. The large phase 3 Lp(a)-HORIZON outcomes trial (NCT04023552) has enrolled about 8,300 patients with established ASCVD and elevated Lp(a) to determine whether lowering Lp(a) translates into reduced cardiovascular events. The trial is expected to report the primary results in 2026.

Lepodisiran is a small-interfering RNA (siRNA) therapeutic that silences the *LPA* gene to inhibit apolipoprotein(a) synthesis. In phase 2 trials, a single or two-dose regimen produced durable Lp(a) reductions of up to 90% or greater, lasting several months [66]. The ongoing phase 3 ACCLAIM-Lp(a) outcomes trial (NCT06292013) is evaluating whether the Lp(a) lowering reduces cardiovascular risk in adults with ASCVD or at high cardiovascular risk. The long duration of action of lepodisiran may enable infrequent dosing.

Olpasiran is a GalNAc-conjugated siRNA that targets apolipoprotein(a) mRNA, achieving reductions in Lp(a) of greater than 95% in the phase 2 OCEAN(a)-DOSE trial [67]. These effects were durable, persisting for months after treatment cessation. The ongoing phase 3 OCEAN(a)-Outcomes trial (NCT05581303) is enrolling patients with established ASCVD and elevated Lp(a) to evaluate the impact of olpasiran on major cardiovascular events. Thus far, olpasiran has shown excellent tolerability, with adverse event rates comparable to placebo.

Zerlasiran is another GalNAc-conjugated siRNA targeting hepatic *LPA* expression, leading to reductions of more than 80% in circulating Lp(a) over 36 weeks in early clinical studies [68]. It was well tolerated, with mainly mild injection-site reactions. A long-term cardiovascular outcomes trial is being planned following early-phase development to confirm safety and assess whether Lp(a) lowering with zerlasiran improves major cardiovascular outcomes.

Muvalaplin represents a first-in-class oral small-molecule inhibitor that disrupts the covalent bonding between apolipoprotein(a) and apolipoprotein B100, preventing formation of Lp(a) particles. In early-phase trials, once-daily oral muvalaplin achieved up to 65–85% reductions in Lp(a) levels with good tolerability [69]. An outcomes study (NCT07157774) is now planned to test whether this novel oral therapy can reduce cardiovascular events in patients with elevated Lp(a) and established ASCVD or at high risk.

Together, these five therapies – pelacarsen, lepodisiran, olpasiran, zerlasiran, and muvalaplin – represent a new generation of Lp(a)-lowering strategies, each employing distinct mechanisms to address a previously untreatable contributor to residual cardiovascular risk.

### Triglyceride-Lowering Drugs

#### Emerging ANGPTL3-Targeting Therapies

Three novel therapies – SHR-1918, zodasiran, and solbinsiran – are advancing as promising treatments for patients with residual lipid abnormalities through inhibition of ANGPTL3, a key regulator of triglyceride and LDL metabolism.

SHR-1918 is a fully human monoclonal antibody that enhances lipase activity and promotes lipid clearance. In a phase 2 trial, it lowered LDL-C by up to ~ 30% and triglycerides by over 50%, with good tolerability [70].

Zodasiran (formerly ARO-ANG3) is a GalNAc-conjugated siRNA that silences hepatic ANGPTL3 expression, producing triglyceride reductions of ~ 50–60% and LDL-C reductions of ~ 20% in phase 2 studies [71]. Its ongoing phase 3 YOSEMITE trial (NCT07037771) is evaluating efficacy in patients with HoFH.

Solbinsiran, another GalNAc-conjugated siRNA targeting ANGPTL3, demonstrated reductions in triglycerides by ~ 50%, LDL-C (~ 17%), and ApoB (~ 14%) in the phase 2 PROLONG-ANG3 study, with an acceptable safety profile [72].

Collectively, these therapies illustrate complementary antibody and RNA interference-based strategies to address residual lipid risk by targeting the ANGPTL3 pathway.

#### Emerging Apolipoprotein CIII-Targeting Therapies

Plozasiran is a GalNAc-conjugated siRNA targeting apoCIII, a key inhibitor of triglyceride and triglyceride-rich lipoprotein (TRL) clearance. In phase 2b and 3 trials, it produced reductions in triglycerides by ~ 60% [73, 74].

Compared to the apoCIII ASO volanesorsen, olezarsen is conjugated with GalNAc resulting in better pharmacology and tolerability. In phase 2 and 3 studies among patients with moderate or severe hypertriglyceridemia and elevated cardiovascular risk, it achieved triglyceride reductions of ~ 50–60% [75–77]. Adverse event rates were similar to placebo and no major safety signals emerged. Olezarsen is positioned to address the unmet need of highly elevated triglycerides and associated cardiovascular and pancreatitis risk.

Together, plozasiran and olezarsen represent a new generation of therapies targeting apoCIII-leveraging RNA-based modalities (siRNA and ASO) to markedly lower triglycerides and related atherogenic lipoproteins, with the goal of reducing events such as acute pancreatitis and cardiovascular outcomes.

### Future Directions

Putting these novel drugs into context, the following high-level future developments beyond specifics of the single compounds may be expected:

An increasing number of evidence-based tools to lower LDL-C to the recommended target concentrations are available, however they remain underutilized worldwide. Cost and restrictions in access to the currently available PCSK9 inhibitors are important reasons for their underutilization. While the production costs of monoclonal antibodies are not expected to undercut a certain threshold, it can be speculated that the production costs of siRNAs such as inclisiran, of the fusion protein lerodalcibep, the macrocyclic peptide enlicitide, and of the oral small-molecule inhibitor laroprovstat might be lower and allow for more attractive pricing in the (distant) future. Oral administration of the two latter compounds may also be advantageous for patients who prefer daily oral dosing over regular injections. With obicetrapib, another mechanistic option may become available for combination therapies. An important perspective are new single-pill combinations, e.g. bempedoic acid with statins and ezetimibe, obicetrapib with ezetimibe, and of oral PCSK9 inhibitors with statins with the potential to offer up to 80% LDL-C reductions with one tablet a day.

Future cardiovascular prevention may undergo a profound shift as novel triglyceride- and Lp(a)-lowering therapies move toward clinical validation. If positive outcomes trials confirm their benefit, targeted interventions for patients with residual ASCVD risk driven by elevated triglycerides or Lp(a) could substantially reduce morbidity. Across dyslipidemia phenotypes, a major therapeutic advance is the emergence of long-acting RNA- and DNA-targeting agents capable of producing sustained reductions in LDL-C, triglycerides, and Lp(a). SiRNA therapeutics targeting PCSK9, ANGPTL3, apoCIII, and Lp(a) already enable dosing intervals of up to six months, potentially improving adherence. Even more transformative are early gene-editing strategies, which suggest that a single infusion could yield durable – potentially lifelong – suppression of pathogenic lipoproteins. Pending confirmation of long-term safety and efficacy, these modalities offer the opportunity to redefine ASCVD prevention, shifting the paradigm from daily treatment to the healing of a disorder with a one-time application.

## Conclusion

The armamentarium to treat dyslipidemia has significantly expanded in the past years. The combination of LDL-C-lowering therapeutics allows for the achievement of the LDL-C targets in the majority of patients, and enables tailored treatment e.g. in patients with statin intolerance. While in patients without ACS, a stepwise approach of treatment is reasonable, ACS patients should be treated with a combination of statin and ezetimibe upfront, preferably as single-pill combination (Fig. 1).

Novel LDL-C-lowering agents are under development, such as alternative PCSK9 inhibitors, in part orally available, and the CETP inhibitor obicetrapib, which have the potential to further improve the utilization of lipid-lowering therapies. In the near future, cardiovascular outcomes trials of specific lipoprotein(a)-lowering agents are expected to be completed, and novel triglyceride-lowering agents have been shown to not only reduce triglyceride concentration, but also the total number of atherogenic lipoproteins, reflected by apolipoprotein B concentration. Positive cardiovascular outcomes trials of these compounds provided, the future of dyslipidemia management may become much more specific than currently, depending on the patient’s dyslipidemia phenotype (Fig. 2; Table 1).

## Key References

- Mach F, Koskinas KC, van Roeters Lennep JE et al. (2025) 2025 Focused Update of the 2019 ESC/EAS Guidelines for the management of dyslipidaemias. Eur Heart J. 10.1093/eurheartj/ehaf190.
  - ○ The 2025 update of the ESC/EAS dyslipidemia guidelines includes novel and revised recommendations based on studies published since the 2019 guidelines.
- Bohula EA, Marston NA, Bhatia AK et al. (2025) Evolocumab in Patients without a Previous Myocardial Infarction or Stroke. N Engl J Med. 10.1056/NEJMoa2514428.
  - ○ In the VESALIUS-CV trial of ~ 12,000 patients without previous myocardial infarction or stroke, treatment with evolocumab reduced the primary endpoint by 25% over a median of 4.6 years of follow-up, providing important information on the efficacy of evolocumab in patients at elevated risk, but without previous cardiovascular event.
- Nissen SE, Lincoff AM, Brennan D et al. (2023) Bempedoic Acid and Cardiovascular Outcomes in Statin-Intolerant Patients. N Engl J Med 388:1353–1364. 10.1056/NEJMoa2215024.
  - ○ In the CLEAR Outcomes trial of ~ 14,000 patients with statin intolerance, bempedoic acid reduced the primary endpoint by 13% without an increase in muscle-related adverse effects.
- Das Pradhan A, Glynn RJ, Fruchart J-C et al. (2022) Triglyceride Lowering with Pemafibrate to Reduce Cardiovascular Risk. N Engl J Med 387:1923–1934. 10.1056/NEJMoa2210645.
  - ○ In the PROMINENT study of ~ 10,500 patients with type 2 diabetes, elevated triglycerides, and low HDL cholesterol, the highly potent and selective PPARα modulator pemafibrate reduced triglycerides by 26% and increased apolipoprotein B by 5%. No cardiovascular benefit was shown.
- Kronenberg F, Mora S, Stroes ESG et al. (2022) Lipoprotein(a) in atherosclerotic cardiovascular disease and aortic stenosis: a European Atherosclerosis Society consensus statement. Eur Heart J 43:3925–3946. 10.1093/eurheartj/ehac361.
  - ○ This consensus document provides a state-of-the-art overview on lipoprotein(a).
- Leosdottir M, Schubert J, Brandts J et al. (2025) Early Ezetimibe Initiation After Myocardial Infarction Protects Against Later Cardiovascular Outcomes in the SWEDEHEART Registry. J Am Coll Cardiol 85:1550–1564. 10.1016/j.jacc.2025.02.007.
  - ○ This analysis based on the large SWEDEHEART registry showed reduced cardiovascular risk for early compared to delayed or no ezetimibe treatment.
- Cuchel M, Raal FJ, Hegele RA et al. (2023) 2023 Update on European Atherosclerosis Society Consensus Statement on Homozygous Familial Hypercholesterolaemia: new treatments and clinical guidance. Eur Heart J 44:2277–2291. 10.1093/eurheartj/ehad197.
  - ○ This is a recent consensus state on homozygous familial hypercholesterolemia.
- Alexander VJ, Karwatowska-Prokopczuk E, Prohaska TA et al. (2024) Volanesorsen to Prevent Acute Pancreatitis in Hypertriglyceridemia. N Engl J Med 390:476–477. 10.1056/NEJMc2306575.
  - ○ This meta-analysis demonstrated a significant reduction in the incidence of pancreatitis in patients with familial chylomicronemia syndrome treated with volanesorsen.

## Data Availability

No datasets were generated or analysed during the current study.
